# Violence Victimization, Substance Use, and Suicide Risk Among Sexual Minority High School Students — United States, 2015–2017

**DOI:** 10.15585/mmwr.mm6743a4

**Published:** 2018-11-02

**Authors:** Michelle M. Johns, Richard Lowry, Catherine N. Rasberry, Richard Dunville, Leah Robin, Sanjana Pampati, Deborah M. Stone, Laura M. Mercer Kollar

**Affiliations:** ^1^Division of Adolescent and School Health, National Center for HIV/AIDS, Viral Hepatitis, STD, and TB, CDC; ^2^Oak Ridge Institute for Science and Education, Oak Ridge, Tennessee; ^3^Division of Violence Prevention, National Center for Injury Prevention and Control, CDC.

Youths identifying as lesbian, gay, bisexual, or another nonheterosexual identity (sexual minority youths) report more violence victimization, substance use, and suicide risk than do heterosexual youths (*1*). These disparities are generally attributed to minority stress (the process through which stigma directed toward sexual minorities influences health outcomes) (*2*,*3*). Sexual minority youths might experience negative outcomes associated with minority stress differently across sexual identities, but to date, no nationally representative study has examined differences in victimization, substance use, and suicide risk within sexual minority youth. Using pooled data from the 2015 and 2017 national Youth Risk Behavior Surveys (YRBS), relationships between sexual identity groups and victimization, substance use, and suicide risk were evaluated with sex-stratified logistic regression models. Compared with heterosexual students, bisexual females and all sexual minority males reported more victimization; lesbian and bisexual females reported more use of alcohol, cigarettes, and marijuana; and all sexual minority youths reported elevated high-risk substance use and suicide risk. Programmatic efforts to reduce and prevent victimization, substance use, and suicide risk among sexual minority youths might benefit from consideration of issues within group differences.

Analyses use pooled data from the 2015 and 2017 cycles of the national YRBS, a biennial, school-based survey of U.S. high school students. YRBS uses a three-stage cluster sample design to select a nationally representative sample of students in grades 9–12 attending public and private schools (*4*). The overall response rate for the 2015 and 2017 YRBS was 60% (both years), and the sample sizes were 15,624 and 14,765, respectively. The combined analytic sample included 30,389 students. Data were weighted to yield nationally representative estimates. Survey procedures protected students’ privacy through anonymous/voluntary participation, using local parental permission procedures. CDC’s Institutional Review Board approved data collection.

Students were grouped into one of four sexual identity categories (gay/lesbian, bisexual, not sure, and heterosexual) based on their response to “Which of the following best describes you?” Seven items assessed victimization: 1) felt unsafe at or traveling to or from school in past 30 days; 2) ever forced to have sexual intercourse; and in past 12 months, 3) threatened or injured with a weapon at school; 4) experienced sexual dating violence; 5) experienced physical dating violence; 6) was bullied at school; and 7) was electronically bullied. Three items assessed lifetime substance use of cigarettes, alcohol, and marijuana, and five items assessed lifetime high-risk substance use (cocaine, heroin, methamphetamines, ecstasy, and inhalants). Five items assessed suicide risk in the past 12 months: 1) felt sad or hopeless, 2) considered attempting suicide, 3) made a suicide plan, 4) attempted suicide, and 5) had a suicide attempt treated by a doctor or nurse.

Analyses used statistical software to account for complex sampling design. Unadjusted prevalence estimates with 95% confidence intervals (CIs) were calculated using Taylor series linearization. Sex-stratified logistic regression models, controlling for race/ethnicity and school grade produced adjusted prevalence ratios (APRs) with heterosexual students serving as the referent group. Models were tested for effect modification by sex (i.e., Wald F statistic for interaction between sex and sexual identity) to determine if associations between sexual identity and victimization, substance use, and suicide risk varied by sex. Post-hoc linear contrast t-tests were used to assess additional between-group differences in prevalence of outcomes across sexual identity. Statistical tests were considered significant if p-values were <0.05 or 95% CIs did not include 1.0.

Compared with heterosexual females, bisexual females reported a higher prevalence of feeling unsafe at or traveling to or from school (APR = 1.6), being threatened/injured with a weapon (2.2), having experienced forced sex (2.8), sexual dating violence (1.7), physical dating violence (1.9), bullying at school (1.7), and electronic bullying (2.3) (Table 1). Bisexual females were more likely than were lesbians to report sexual dating violence, and more likely than both lesbian and females not sure of their sexual identity to experience forced sex, bullying at school, and electronic bullying. Males who were gay, bisexual, or not sure of their sexual identity had a higher likelihood than did heterosexual males of reporting all seven indicators of violence victimization. Prevalence estimates and significant effect modifications by sex indicate that associations between sexual minority status and victimization were stronger for males than for females for experiencing forced sex, sexual dating violence, physical dating violence, being bullied at school, and being electronically bullied.

**TABLE 1:** Unadjusted prevalence and adjusted prevalence ratios of experiences of violence victimization, by sex and sexual identity — National Youth Risk Behavior Survey, United States, 2015–2017*

Experience/Sex	Heterosexual^†^	Lesbian/Gay		Bisexual		Not sure		Interaction by sex p-value
	% (95% CI)	% (95% CI)	APR (95% CI)	% (95% CI)	APR (95% CI)	% (95% CI)	APR (95% CI)	
**Felt unsafe at or traveling to/from school^§^**								
Females	5.8 (5.0–6.7)	11.0 (7.5–15.9)	1.9^¶^ (1.3–2.9)	9.6 (8.0–11.6)	1.6^¶^ (1.3–2.0)	9.4 (6.7–13.0)	1.6^¶^ (1.1–2.2)	0.135
Males	4.7 (4.2–5.4)	18.0 (10.9–28.2)	3.7^¶^ (2.4–5.8)	10.6 (6.9–15.8)	2.4^¶^ (1.6–3.7)	11.2 (7.6–16.2)	2.3^¶^ (1.5–3.5)	
Threatened or injured with a weapon at school**								
Females	3.7 (3.2–4.3)	5.9 (3.3–10.3)	1.5 (0.8–2.7)	8.6 (6.9–10.7)	2.2^¶^ (1.7–2.8)	6.1 (3.8–9.8)	1.6 (1.0–2.7)	0.210
Males	6.5 (5.9–7.2)	15.1 (9.3–23.6)	1.9^¶^ (1.2–2.8)	11.6 (7.6–17.5)	1.8^¶^ (1.2–2.8)	17.2 (12.5–23.1)	2.5^¶^ (1.8–3.4)	
**Experienced forced sexual intercourse^††^**								
Females	8.8 (7.7–10.1)	15.4 (11.3–20.6)	1.7^¶,§§^ (1.2–2.4)	23.9 (21.2–26.8)	2.8^¶,¶¶^ (2.4–3.2)	11.4 (8.0–16.2)	1.3 (0.9–1.8)	0.000
Males	2.5 (2.1–2.9)	17.1 (11.2–25.3)	6.6^¶^ (4.4–9.8)	8.1 (4.7–13.4)	3.3^¶^ (2.0–5.6)	12.6 (8.9–17.4)	4.7^¶^ (3.1–7.0)	
Experienced sexual dating violence***								
Females	12.0 (10.9–13.3)	10.8 (7.1–16.0)	1.0^§§^ (0.6–1.5)	20.8 (17.5–24.6)	1.7^¶^ (1.4–2.1)	18.0 (13.1–24.2)	1.5^¶^ (1.1–2.1)	0.000
Males	3.3 (2.8–3.9)	24.0 (13.1–39.7)	5.5^¶^ (3.0–10.1)	12.6 (7.6–20.1)	3.9^¶^ (2.3–6.6)	14.7 (9.6–22.0)	4.5^¶^ (2.8–7.2)	
**Experienced physical dating violence^†††^**								
Females	9.0 (7.9–10.2)	13.8 (9.1–20.5)	1.5 (0.9–2.3)	17.5 (15.0–20.2)	1.9^¶^ (1.6–2.3)	13.6 (10.0–18.2)	1.5^¶^ (1.1–2.1)	0.008
Males	6.0 (5.4–6.7)	24.2 (14.6–37.3)	3.2^¶^ (2.0–4.9)	14.5 (8.5–23.8)	2.5^¶^ (1.4–4.2)	21.6 (14.6–30.9)	3.3^¶^ (2.1–5.2)	
**Bullied at school** ^§§§^								
Females	21.9 (20.5–23.5)	25.6 (19.5–32.8)	1.3^§§^ (1.0–1.7)	36.1 (32.0–40.4)	1.7^¶,¶¶^ (1.5–1.8)	22.6 (18.8–26.9)	1.0 (0.9–1.2)	0.001
Males	14.6 (13.7–15.5)	27.7 (20.1–36.9)	2.0^¶^ (1.5–2.8)	33.5 (27.1–40.7)	2.3^¶^ (1.8–2.8)	26.2 (21.3–31.7)	1.8^¶^ (1.4–2.2)	
**Electronically bullied^¶¶¶^**								
Females	19.6 (18.4–20.9)	21.4 (15.9–28.1)	1.2^§§^ (0.9–1.6)	30.9 (27.3–34.8)	1.6^¶,¶¶^ (1.4–1.8)	22.1 (17.8–27.1)	1.2 (0.9–1.4)	0.000
Males	8.8 (8.0–9.6)	17.7 (12.6–24.3)	1.9^¶,¶¶^ (1.3–2.7)	26.3 (20.6–32.8)	3.0^¶^ (2.3–3.9)	20.1 (15.4–25.8)	2.2^¶^ (1.7–3.0)	

**Abbreviations:** APR = adjusted (for race/ethnicity and grade) prevalence ratio; CI = confidence interval.

* Statistical significance is indicated when p<0.05 or 95% CI does not include 1.0.

^†^ Referent group is heterosexual students.

^§^ Did not go to school because they felt unsafe at school or on their way to or from school on at least 1 day during the 30 days before the survey.

^¶^ Significantly different from heterosexual students.

** Threatened or injured with a weapon on school property during the 12 months before the survey.

^††^ Ever physically forced to have sexual intercourse when they did not want to.

^§§^ Significantly different from bisexual students.

^¶¶^ Significantly different from students who are not sure of their sexual identity.

*** Being forced to do sexual things they did not want to do by someone they were dating or going out with one or more times during the 12 months before the survey, among students who dated or went out with someone during the 12 months before the survey.

^†††^ Being physically hurt on purpose by someone they were dating or going out with one or more times during the 12 months before the survey, among students who date or went out with someone during the 12 months before the survey.

^§§§^ Bullied on school property during the 12 months before the survey.

^¶¶¶^ Bullied through texting, Instagram, Facebook, or other social media during the 12 months before the survey.

Lesbians reported a higher prevalence of using cigarettes (APR = 1.8) and marijuana (1.5) than did heterosexual females (Table 2). Compared with heterosexual females, bisexual females had a higher prevalence of use of cigarettes (1.8), alcohol (1.2), and marijuana (1.6); females who were not sure of their sexual identity reported a higher prevalence of using cigarettes (1.2), but a lower prevalence of using alcohol (0.9). The prevalences of reported use of cocaine, heroin, methamphetamines, ecstasy, and inhalants were higher among lesbian, bisexual, and females not sure of their sexual identity than among heterosexual females. Among male students, compared with those identifying as heterosexual, bisexuals had a higher prevalence of reported cigarette use (1.4), and those identifying as not sure had a lower reported prevalence of marijuana use (0.8). The reported prevalences of using cocaine, heroin, methamphetamines, ecstasy, and inhalants were higher among gay and bisexual males and males who were not sure of their sexual identity than among heterosexual males. Prevalence estimates and significant effect modifications by sex indicate that associations between sexual minority status and cigarettes/marijuana use were stronger for females than for males.

**TABLE 2:** Unadjusted prevalence and adjusted prevalence ratios of lifetime substance use behaviors by sex and sexual identity — National Youth Risk Behavior Survey, United States, 2015–2017*

Substance use behavior/Sex	Heterosexual^†^	Lesbian/Gay		Bisexual		Not sure		Interaction by sex p-value
	% (95% CI)	% (95% CI)	APR (95% CI)	% (95% CI)	APR (95% CI)	% (95% CI)	APR (95% CI)	
**Cigarettes**								
Females	26.4 (23.7–29.4)	46.8 (39.3–54.3)	1.8^§,¶^ (1.5–2.1)	47.1 (42.6–51.7)	1.8^§,¶^ (1.6–2.0)	32.1 (26.4–38.5)	1.2^§^ (1.0–1.5)	0.007
Males	32.1 (29.9–34.4)	38.4 (29.9–47.8)	1.2 (1.0–1.5)	43.8 (37.4–50.4)	1.4^§,¶^ (1.2–1.6)	32.4 (26.3–39.1)	1.0 (0.8–1.2)	
**Alcohol**								
Females	63.8 (61.4–66.2)	68.1 (61.3–74.2)	1.1^§,¶^ (1.0–1.2)	78.1 (74.3–81.4)	1.2^§,¶^ (1.2–1.3)	54.9 (48.4–61.2)	0.9^§^ (0.8–1.0)	0.064
Males	60.2 (58.6–61.7)	64.2 (55.2–72.3)	1.1 (0.9–1.2)	66.5 (58.3–73.9)	1.1^¶^ (1.0–1.2)	55.1 (47.8–62.1)	0.9 (0.8–1.1)	
**Marijuana**								
Females	34.5 (32.1–37.0)	55.1 (47.3–62.7)	1.5^§,¶^ (1.3–1.8)	55.6 (51.9–59.2)	1.6^§,¶^ (1.5–1.8)	36.0 (30.2–42.2)	1.1 (0.9–1.3)	0.000
Males	38.1 (35.9–40.3)	43.5 (34.2–53.3)	1.1^¶^ (0.9–1.4)	37.4 (29.6–45.9)	1.0 (0.8–1.2)	31.3 (25.8–37.3)	0.8^§^ (0.7–1.0)	
**Cocaine**								
Females	3.0 (2.6–3.5)	6.4 (4.2–9.7)	2.2^§^ (1.4–3.4)	6.3 (5.0–7.9)	2.2^§^ (1.6–2.9)	6.5 (4.2–9.8)	2.2^§^ (1.4–3.4)	0.069
Males	5.2 (4.5–5.9)	21.4 (13.7–31.8)	3.6^§^ (2.5–5.0)	12.1 (7.5–18.9)	2.5^§^ (1.5–4.1)	17.6 (12.7–23.7)	3.0^§^ (2.1–4.4)	
**Heroin**								
Females	0.7 (0.5–1.0)	4.3 (2.4–7.6)	5.8^§^ (3.0–11.1)	2.2 (1.5–3.2)	3.0^§^ (1.9–4.8)	2.8 (1.4–5.8)	3.9^§^ (1.8–8.6)	0.169
Males	1.7 (1.3–2.1)	13.4 (7.2–23.7)	6.1^§^ (4.1–9.3)	8.1 (4.4–14.3)	5.2^§^ (3.0–9.1)	14.4 (10.3–19.7)	8.1^§^ (5.5–11.9)	
**Methamphetamines**								
Females	1.2 (1.0–1.6)	6.4 (3.4–11.9)	4.9^§^ (2.5–9.7)	4.4 (3.2–5.9)	3.6^§^ (2.4–5.3)	3.8 (2.3–6.2)	2.8^§^ (1.6–5.1)	0.334
Males	2.5 (2.1–3.0)	18.7 (10.7–30.6)	6.2^§^ (4.0–9.6)	9.4 (5.6–15.2)	3.9^§^ (2.3–6.6)	14.4 (9.7–20.9)	5.2^§^ (3.3–8.1)	
**Ecstasy**								
Females	2.7 (2.2–3.3)	4.8 (3.0–7.4)	1.8^§^ (1.1–3.0)	7.3 (5.9–9.0)	2.8^§^ (2.1–3.7)	5.4 (3.5–8.4)	2.0^§^ (1.2–3.3)	0.088
Males	4.6 (4.0–5.3)	15.9 (10.7–23.0)	3.2^§^ (2.2–4.7)	15.4 (10.6–21.8)	3.6^§^ (2.4–5.3)	16.4 (11.3–23.3)	3.5^§^ (2.3–5.3)	
**Inhalants**								
Females	5.2 (4.6–6.0)	11.3 (7.3–16.9)	2.3^§^ (1.4–3.5)	12.5 (10.3–15.1)	2.4^§^ (1.9–2.9)	13.8 (9.6–19.4)	2.6^§^ (1.8–3.7)	0.176
Males	5.5 (5.0–6.1)	20.0 (11.7–32.1)	3.2^§^ (2.1–5.0)	14.9 (10.5–20.7)	2.8^§^ (1.9–4.0)	22.2 (17.1–28.2)	3.9^§^ (2.9–5.1)	

**Abbreviations:** APR = adjusted (for race/ethnicity and grade) prevalence ratio; CI = confidence interval.

* Statistical significance is indicated when p<0.05 or 95% CI does not include 1.0.

^†^ Referent group is heterosexual students.

^§^ Significantly different from heterosexual students.

^¶^ Significantly different from students who are not sure of their sexual identity.

** Significantly different from bisexual students.

All sexual minority females reported a higher prevalence of feeling sad or hopeless, considering attempting suicide, making a suicide plan, and attempting suicide than did heterosexual females (Table 3). The reported prevalence of a suicide attempt treated by a doctor or nurse was higher among lesbian (APR = 3.7) and bisexual females (3.7) than among heterosexual females. Compared with heterosexual males, all sexual minority males had higher prevalences of all five indicators of suicide risk. Prevalence estimates and significant effect modification indicate that the association between sexual identity and attempted suicide was stronger for males than for females.

**TABLE 3:** Unadjusted prevalence and adjusted prevalence ratios of suicide risk behaviors by sex and sexual identity — National Youth Risk Behavior Survey, United States, 2015–2017

Suicide risk behaviors/Sex	Heterosexual*	Lesbian/Gay		Bisexual		Not sure		Interaction by sex p-value
	% (95% CI)	% (95% CI)	APR (95% CI)	% (95% CI)	APR (95% CI)	% (95% CI)	APR (95% CI)	
**Sad/Hopeless^†^**								
Females	36.1 (34.0–38.3)	59.4 (51.2–67.2)	1.7 ^§,¶,^** (1.5–1.9)	69.3 (66.1–72.4)	1.9^§,¶^ (1.8–2.1)	51.0 (44.7–57.2)	1.4^§^ (1.3–1.6)	0.385
Males	19.0 (17.9–20.2)	39.4 (32.3–47.0)	2.0^§^ (1.7–2.5)	49.1 (41.7–56.5)	2.6^§,¶^ (2.2–3.1)	38.3 (32.2–44.8)	2.0^§^ (1.6–2.4)	
**Considered attempting suicide^††^**								
Females	18.3 (17.2–19.5)	45.8 (37.7–54.2)	2.6^§,¶^ (2.1–3.1)	49.7 (46.0–53.3)	2.7^§,¶^ (2.5–3.0)	34.5 (28.6–40.8)	1.9^§^ (1.6–2.2)	0.242
Males	10.4 (9.7–11.2)	28.0 (20.7–36.8)	2.7^§,¶^ (2.0–3.7)	40.6 (32.9–48.8)	4.0^§,¶^ (3.3–4.8)	27.1 (22.1–32.7)	2.6^§^ (2.1–3.2)	
**Made a suicide plan^§§^**								
Females	14.3 (13.1–15.6)	38.0 (30.6–46.0)	2.7^§,¶^ (2.2–3.3)	42.0 (38.5–45.6)	2.9^§,¶^ (2.6–3.3)	27.9 (23.2–33.2)	1.9^§^ (1.6–2.3)	0.330
Males	8.4 (7.7–9.3)	23.3 (17.8–29.7)	2.6^§,¶^ (2.0–3.5)	31.7 (24.7–39.6)	3.9^§,¶^ (3.0–5.0)	22.6 (17.8–28.3)	2.7^§^ (2.1–3.5)	
**Attempted suicide^¶¶^**								
Females	7.7 (6.7–8.9)	24.3 (17.4–32.8)	3.2^§,¶^ (2.3–4.5)	28.3 (24.7–32.3)	3.6^§,¶^ (3.0–4.2)	12.4 (9.5–16.1)	1.5^§^ (1.1–2.1)	0.012
Males	4.3 (3.8–4.9)	13.1 (7.7–21.2)	3.0^§,††^ (1.9–4.9)	23.2 (17.0–30.7)	5.5^§,¶^ (4.1–7.5)	14.9 (10.9–20.0)	3.3^§^ (2.3–4.8)	
Suicide attempt treated by a doctor or nurse***								
Females	2.4 (1.9–3.0)	8.7 (4.6–15.7)	3.7^§^ (1.9–7.1)	9.2 (7.4–11.4)	3.7^§,¶^ (2.8–5.0)	4.2 (2.4–7.0)	1.6 (0.9–2.9)	0.545
Males	1.4 (1.1–1.8)	4.9 (2.4–9.6)	3.7^§^ (1.8–7.5)	5.6 (2.7–11.1)	4.2^§^ (2.2–7.9)	5.3 (2.7–10.0)	3.5^§^ (1.6–7.8)	

**Abbreviations:** APR = adjusted (for race/ethnicity and grade) prevalence ratio; CI = confidence interval.

* Referent group.

^†^ Felt sad or hopeless almost every day for ≥2 weeks in a row so that they stopped doing some usual activities during the 12 months before the survey.

^§^ Significantly different from heterosexual students.

^¶^ Significantly different from students who are not sure of their sexual identity.

** Significantly different from bisexual students.

^††^ Seriously considered attempting suicide during the 12 months before the survey.

^§§^ Made a plan about how they would attempt suicide during the 12 months before the survey.

^¶¶^ Attempted suicide one or more times during the 12 months before the survey.

*** Attempted suicide that resulted in an injury, poisoning, or overdose that had to be treated by a doctor or nurse during the 12 months before the survey.

## Discussion

These findings demonstrate that sexual minority youths generally experience disparities in health outcomes attributed to minority stress when compared with heterosexual youths. Furthermore, some critical differences exist among sexual minority youths.

Bisexual female high school students experienced more disparities in victimization than did females who identified as heterosexual, lesbian, or not sure of their sexual identity. This finding differs from research indicating that lesbian and bisexual females experience similar rates of peer victimization (*5*), but supports study findings that bisexual female adults experience heightened risk for sexual violence (*6*). Among males, all sexual minority students were more likely than were heterosexual students to have experienced violence victimization. Associations between sexual minority status and victimization were stronger among males than among females, consistent with previous findings on gender differences among sexual minority youths (*5*). Violence prevention programs might need to consider the unique social stressors faced by bisexual females and sexual minority males.

Lesbian and bisexual female students reported higher levels of use of alcohol, cigarettes, and marijuana than did heterosexuals, whereas few differences across sexual identity for these substances were found among male students. These results are consistent with findings from previous research suggesting that sexual minority females are at increased risk for using alcohol, cigarettes, and marijuana (*7*). All sexual minority students were more likely than were heterosexual students to engage in high-risk substance use (i.e., less prevalent substances with a high risk of adverse outcomes), suggesting a need for increased primary and secondary prevention of high-risk substance use for sexual minority youths.

Suicide risk was higher among sexual minority students, regardless of sex or sexual identity, than among heterosexual students, which is consistent with the broader literature on suicide risk and sexual minority youths (*8*). The ubiquity of elevated APRs for suicidal thoughts and behaviors among sexual minority youths reinforces the important need for suicide prevention programming that is relevant and efficacious for this group.

The findings in this report are subject to at least five limitations. First, data are cross-sectional and indicate only association, not causation. Second, whether “not sure” students were not sure of their identity, the usefulness of the response options for their identity, or the meaning of the question itself is unclear. Third, YRBS responses are self-reported, and might be subject to reporting bias; although the extent of underreporting or overreporting cannot be determined, the survey questions generally demonstrate good test-retest reliability (*4*). Fourth, the national YRBS does not currently ask about gender identity, and thus the prevalence of these outcomes for transgender students cannot be assessed with these data. Finally, CDC collects YRBS data in schools; because students at highest risk for victimization, substance use, and suicide risk might have dropped out, these findings might underestimate the observed associations with sexual minority status (*9*).

These findings serve to highlight the variation in victimization, substance use, and suicide risk among high school students by sexual identity. Prevention efforts might benefit from acknowledging within-group differences among sexual minority youths, and by updating and tailoring intervention strategies accordingly (e.g., the recently released suite of CDC technical packages for violence prevention*). More work is needed to understand whether programs promoting safe and supportive environments in schools and communities or stable, nurturing relationships could be better designed to address the within-group differences among sexual minority youths based on their sexual identity (*10*). Finally, professional development for educators and health providers working with sexual minority youths might benefit from content clearly articulating the differences between lesbian/gay, bisexual, and not sure identities, particularly as they relate to victimization, substance use, and suicide risk (*10*). Future research is necessary to determine the ideal components of such programs.

**Summary**

**What is already known about this topic?**

Sexual minority youths are at increased risk for certain adverse health outcomes.

**What is added by this report?**

Analysis of 2015 and 2017 Youth Risk Behavior Survey data identified within-group differences in victimization, substance use, and suicide risk among sexual minority high school students. Compared with heterosexuals, females who are bisexual and males who are gay, bisexual, or not sure of sexual identity reported more victimization; lesbian and bisexual females reported more alcohol, cigarette, and marijuana use; and all sexual minorities reported elevated high-risk substance use and suicide risk behavior.

**What are the implications for public health practice?**

Consideration of the different experiences of victimization, substance use, and suicide risk by sexual identity might help to inform interventions.

## Footnotes

* https://www.cdc.gov/violenceprevention/pub/technical-packages.html.
